# Ectomycorrhizal fungus supports endogenous rhythmic growth and corresponding resource allocation in oak during various below- and aboveground biotic interactions

**DOI:** 10.1038/s41598-021-03132-y

**Published:** 2021-12-08

**Authors:** Mika T. Tarkka, Thorsten E. E. Grams, Oguzhan Angay, Florence Kurth, Hazel R. Maboreke, Sarah Mailänder, Markus Bönn, Lasse Feldhahn, Frank Fleischmann, Liliane Ruess, Martin Schädler, Stefan Scheu, Silvia D. Schrey, Francois Buscot, Sylvie Herrmann

**Affiliations:** 1 Department of Soil Ecology, UFZ-Helmholtz Centre for Environmental Research, Theodor-Lieser-Str. 4, 06120 Halle (Saale), Germany; 2 German Centre for Integrative Biodiversity Research (iDiv) Halle-Jena-Leipzig, Deutscher Platz 5e, 04103 Leipzig, Germany; 3 Department of Ecology and Ecosystem Science, Plant Ecophysiology, Technische Universität München, Hans-Carl-von-Carlowitz Platz 2, Freising, Germany; 4 Institute of Biology, Ecology Group, Humboldt-Universität Zu Berlin, Philippstraße 13, 10115 Berlin, Germany; 5 Interfaculty Institute of Microbiology and Infection Medicine Tübingen (IMIT), Auf der Morgenstelle 1, 72076 Tübingen, Germany; 6 Landesamt Für Verbraucherschutz Sachsen-Anhalt, Freiimfelder Str. 68, 06112 Halle, Germany; 7 Institut Für Informatik, Martin-Luther-Universität Halle-Wittenberg, 06120 Halle, Germany; 8 Ludwig-Maximilians-University Munich, Chair of Experimental Physics – Laser physics, Am Coulombwall 1, 85748 Garching, Germany; 9 Department of Community Ecology, Helmholtz-Centre for Environmental Research-UFZ, Theodor-Lieser-Strasse 4, 06110 Halle (Saale), Germany; 10 Centre of Biodiversity and Sustainable Land Use, University of Göttingen, Büsgenweg 1, 37077 Göttingen, Germany; 11 J.F. Blumenbach Institute of Zoology and Anthropology, University of Göttingen, Untere Karspüle 2, 37073 Göttingen, Germany; 12 Institute of Bio- and Geosciences, IBG-2: Plant Sciences, Leo- Brandt-Straße, Forschungszentrum Jülich, 52425 Jülich, Germany

**Keywords:** Plant physiology, Plant symbiosis

## Abstract

Endogenous rhythmic growth (ERG) is displayed by many tropical and some major temperate tree species and characterized by alternating root and shoot flushes (RF and SF). These flushes occur parallel to changes in biomass partitioning and in allocation of recently assimilated carbon and nitrogen. To address how biotic interactions interplay with ERG, we cross-compared the RF/SF shifts in oak microcuttings in the presence of pathogens, consumers and a mycorrhiza helper bacterium, without and with an ectomycorrhizal fungus (EMF), and present a synthesis of the observations. The typical increase in carbon allocation to sink leaves during SF did not occur in the presence of root or leaf pathogens, and the increase in nitrogen allocation to lateral roots during RF did not occur with the pathogens. The RF/SF shifts in resource allocation were mostly restored upon additional interaction with the EMF. Its presence led to increased resource allocation to principal roots during RF, also when the oaks were inoculated additionally with other interactors. The interactors affected the alternating, rhythmic growth and resource allocation shifts between shoots and roots. The restoring role of the EMF on RF/SF changes in parallel to the corresponding enhanced carbon and nitrogen allocation to sink tissues suggests that the EMF is supporting plants in maintaining the ERG.

## Introduction

Plants and especially long lived plants such as trees have to permanently adjust the allocation of their resource investment between growth and defence^1^. Beneficial and detrimental biotic interactions on plants affect carbon (C) and nitrogen (N) allocation^2^. For instance, mycorrhizal infection stimulates invertase activity and sink strength in roots^3^, and leaf infection by a fungal pathogen induces a sink at the point of infection^4^. Alternatively, resources can be transported away from the site of attack. Assimilate transport to roots was increased after herbivory of *Nicotiana attenuata* leaves^5^, and as a response to root herbivory, *Centaurea maculosa* shifted more of the acquired N to shoots to maintain shoot N status and biomass^6^. Investigations on resource allocation between growth and defence, and the mechanisms behind mostly ignore that besides detrimental interactions, most plants are engaged in beneficial interactions such as mycorrhizal symbioses, which not only trigger resource acquisition from soil and via enhanced photoassimilation, but also interfere with pathogen or herbivore interactions^7^.

Many tropical and also some important temperate forest trees display a rhythmic growth pattern characterized by successive periods of growth and rest that alternate between shoots and roots and affect plant architecture^8^. For temperate trees such as oaks, rhythmic growth is an endogenous determined process, which is expressed under controlled conditions providing long days and an optimal temperature regime^9^. Under natural conditions, rhythmic growth is modulated by seasonal variations of environmental variables^10^. Older oak seedlings and stump sprouts typically display an alternation of root growth flushes (RF) with shoot flushes (SF)^11^. Compared to other forest trees, oaks gather especially large communities of insects, and engage symbiosis with a wide variety of mycorrhizal fungi^12,13^. In a microcosm system with microcuttings of *Quercus robur* clone DF159 displaying an endogenous rhythmic growth (ERG) under optimal culture conditions^14^, both carbon and to a lesser extent nitrogen are allocated to the proliferating roots during RF and expanding leaves during SF^15^. Inoculation with the ectomycorrhizal fungus (EMF) *Piloderma croceum* increases both the amplitude of such shifts in resource allocation (RA) between RF (high RA to roots) and SF (high RA to shoots) and the above- and belowground plant development, without altering the period of the ERG cycles. Herrmann et al*.*^15^ also showed that the described ERG relates to changes in plant gene regulation patterns. Furthermore, the ERG in oaks was shown to affect the abundance of ectomycorrhizal roots tips and their distribution pattern on the mother roots^16^. Consumers of oak roots and leaves affect the allocation of carbon and nitrogen in a different manner than the EMF. Interaction with root consuming Collembola (*Protaphorura armata*) reduces the relative growth rate of oak independently of ERG stage and presence of the EMF. By stable isotope labelling experiments it was shown that this leads to an increased ^13^C and ^15^N excess in aboveground plant compartments, and by gene expression analysis it was detected that the expression of growth related genes is triggered in leaves^17^. With the same methods it was shown that the aboveground herbivory by gypsy moth (*Lymantria dispar*) reduces ^13^C and ^15^N allocation to sink leaves, but enhances ^15^N allocation to source leaves, accompanied by an enhanced expression of genes related to the transport of carbohydrates in leaves^18^. In contrast to the herbivores, mycorrhiza helper bacterium causes a complex, not entirely beneficial or detrimental response in the oak system. It stimulates mycorrhiza formation by the EMF and counteracts the damage by parasitic nematodes^19^ and oak powdery mildew^20^, but, when inoculated alone, it causes systemic stress associated transcriptional response in oak^21^. It has not yet been substantiated how interacting organisms modify or support the developmental pattern of resource allocation during ERG, and all these findings open up the question whether and how biotic interactions other than EMF affect the shifts in resource allocation between RF and SF.

To address this question, oak microcuttings were confronted with four interacting organisms, representing the root pathogenic oomycete *Phytophthora quercina* (root pathogen) and the ascomycete leaf powdery mildew *Microsphaera alphitoides* (mildew), the consumer, plant root parasitic nematode *Pratylenchus penetrans* (root parasite), as well as the mycorrhiza helper bacterium *Streptomyces* sp. AcH505 (mycorrhiza helper). C and N allocation was measured at the plant organ level. Co-inoculation with *P. croceum* was also included, to assess if it further modifies the allocation shifts during ERG. Five hypotheses were tested. (1) Interactions with detrimental organisms induce a local sink for resources to support defence, and this plant response alters the RF/SF allocation patterns during the ERG. (2) EMF is able to restore such an alteration of the RF/SF related resource allocation pattern. A third hypothesis is based on a previous result of Herrmann et al*.*^15^, who showed that among the organs of oak microcuttings, C allocation during ERG to principal roots is most significantly affected by the EMF. (3) We thus hypothesized, that the restoration of RF/SF biomass partitioning and resource allocation by the EMF are expressed particularly strongly in principal roots. (4) We hypothesised that the stem is not affected by RF/SF related changes of resource allocation but it does respond to the EMF by a stimulated resource transport between roots and shoots. Based on our earlier research on oak gene expression responses to mycorrhiza helper and root parasite^21,22^, we expected that the changes in RF/SF related resource allocation pattern are also reflected in oak gene expression during interactions with the two pathogens, *Phytophthora* on root and mildew on leaves. We expected (5) an induction of defence related gene expression by the pathogens, but an attenuation of this response by additional EMF interaction.

## Results

### Biomass partitioning and resource allocation in oak microcuttings during biotic interactions

**Figure 1:**
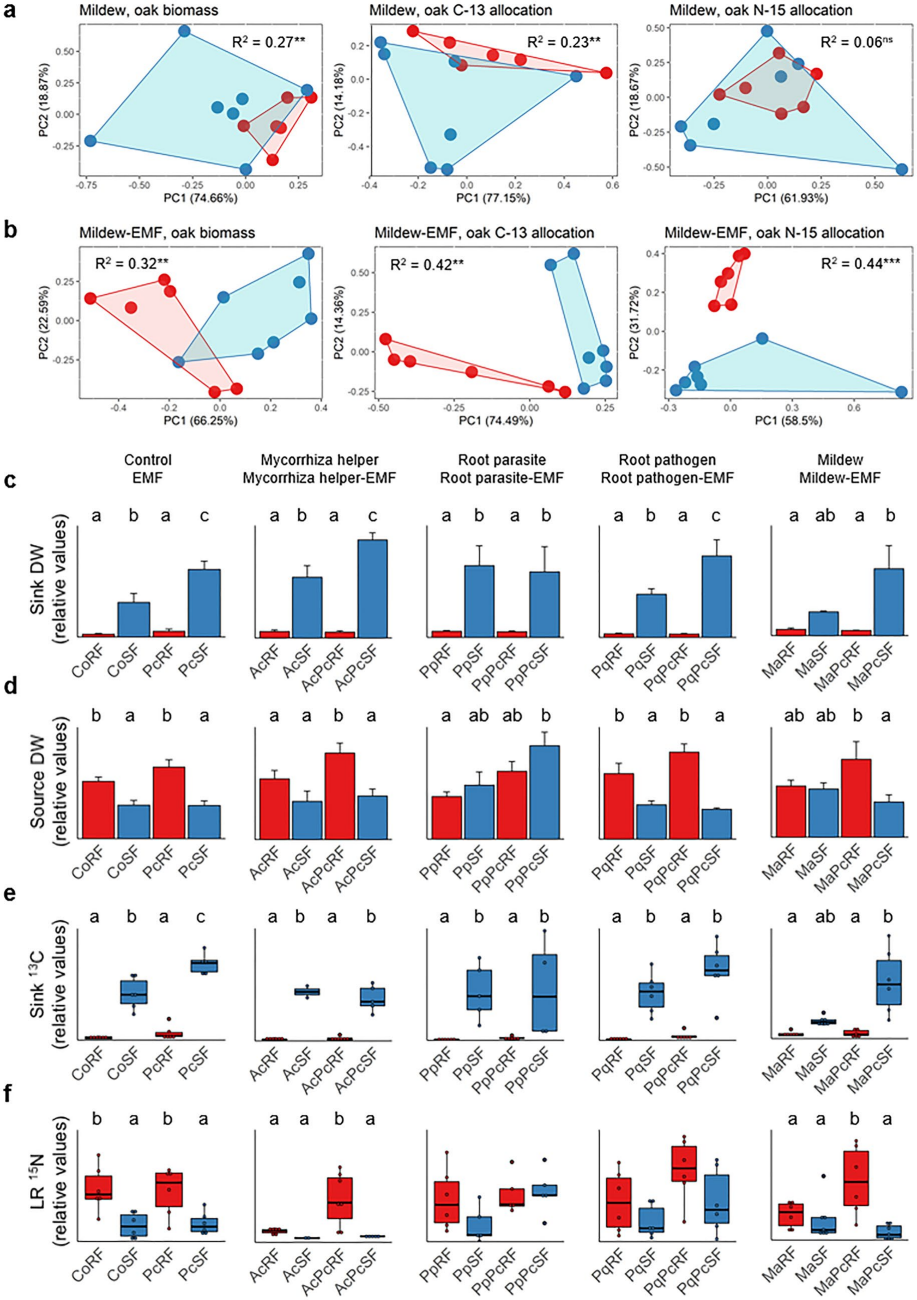
Developmental stage dependent distribution of biomass and resource allocation in oak with biotic interactions. (**a, b**) Principal components analysis of the combined variables of sink and source leaves, stems, principal and lateral roots in mildew treatment (Ma) and in mildew and additional mycorrhizal fungal treatment (MaPc) during root flush (RF) and shoot flush (SF). Each symbol represents the total distribution of resources in an oak individual. Data points for each group are enclosed with a line, and red colour marks RF and blue colour marks SF. Based on a permutational multivariate analysis of variance, R-squared (R^2^) represents the proportion of the variance that's explained by growth stage, and asterisks indicate significant differences between the growth stages at the significance levels ***(*P* < 0.001); **(*P* < 0.01); *(*P* < 0.05); ns (not significant). (**c**–**f**) Extent of biomass, recently fixed carbon and nitrogen (excess of ^13^C and excess of ^15^N) to the organs of oak during root and shoot flush in oak microcuttings engaged in biotic interactions. For the purpose of simplicity, relative values are shown here, and absolute values are given in Supplementary Fig. S3. Only those plant organs are shown, where the extent of resources changed from root flush to shoot flush in controls. Different letters indicate a significant difference according to ANOVA and Tukey HSD test (*P* < 0.05). The boxplots include a line marking the median, middle box representing the middle 50% of scores for each treatment, as well as whiskers and dots representing the scores outside and strongly deviating from the middle 50% of the scores. Please refer to Supplementary Table S4 for the values of difference. Where no lettering is included, the treatments were not different to each other. DW, dry weight; ^13^C, allocation of C-13; ^15^N, allocation of N-15; RF, root flush; SF, shoot flush; Pc, *Piloderma croceum*, Ac, *Streptomyces* sp. AcH505; AcPc = *Streptomyces* sp. AcH505 and *Piloderma croceum*; Pp, *Pratylenchus penetrans*, Pq, *Phytophthora quercina*; Ma, *Microsphaera alphitoides.*

Biomass partitioning and allocation of carbon (C) and nitrogen (N) of oak microcuttings under biotic interactions were evaluated during two growth stages, root flush (RF) and shoot flush (SF), in source and sink leaves, stems, and principal and lateral roots. PERMANOVA indicated significant growth flush dependent (RF/SF) differences between biomass partitioning, as well as C and N allocation in the microcuttings (Supplementary Table S3; example visualisation of mildew and mildew-EMF interactions by principal components analysis in Fig. 1a, b, and visualisations of other interactions in Supplementary Fig. S2). The biomass partitioning RF/SF difference was highly significant without biotic interaction (R^2^ = 0.32, *P* = 0.01), with ectomycorrhizal fungus (EMF, R^2^ = 0.41, *P* = 0.002), with root pathogen (R^2^ = 0.31, *P* = 0.009) and with mildew (R^2^ = 0.27, *P* = 0.004, Fig. 1a). The difference was only marginally significant with mycorrhiza helper (R^2^ = 0.29, *P* = 0.034), but no significant difference was detected with the root parasite. RF/SF difference in C allocation was highly significant in control plants (R^2^ = 0.40, *P* = 0.009), with EMF (R^2^ = 0.44, *P* = 0.004), mildew (R^2^ = 0.32, *P* = 0.001, Fig. 1b) and root pathogen, (R^2^ = 0.315, *P* = 0.002), but not with root parasite or mycorrhiza helper (Supplementary Table S3, Supplementary Fig. S2). By contrast, this difference was restored in root parasite-EMF (R^2^ = 0.28, *P* = 0.005) and mycorrhiza helper-EMF interactions (R^2^ = 0.52, *P* = 0.01). The RF/SF difference in N allocation was significant with control (R^2^ = 0.32, *P* = 0.006), EMF (R^2^ = 0.38, *P* = 0.008), root pathogen (R^2^ = 0.32, *P* = 0.001) and mycorrhiza helper (R^2^ = 0.49: *P* = 0.019), but not in the root parasite and mildew interactions (Supplementary Table S3, Supplementary Fig. S2). Again, the RF/SF differences of N allocation emerged upon co-treatment with EMF, with mildew-EMF (R^2^ = 0.44, *P* = 0.001, Fig. 1b) and root parasite-EMF (R^2^ = 0.21, *P* = 0.029) (Supplementary Table S3; Supplementary Fig. S2). These results show that plant inoculation with mycorrhiza helper, root pathogen, root parasite or mildew, in part, suppress the amplitude of RF/SF changes, and that additional interaction with EMF may restore it.

### Biomass partitioning and resource allocation to leaves and roots: dependence on growth stage as modified by biotic interactions

The differences in biomass partitioning and C and N allocation between RF and SF were related to alternating patterns in distinct plant organs (Fig. 1c–f; Supplementary Table S4; Supplementary Fig. S3). Sink leaf dry weight was lower during RF than SF in the control treatment (Fig. 1c). This reflects the fact that swelling buds during RF are compared to expanded leaves during SF. The difference in partitioning and C and N allocation between RF and SF was present in all interactions with the exception of the one with mildew, but it did occur with mildew and EMF (ANOVA and Tukey’s test; *P* = 0.011). In the controls, source leaf dry weight was higher during RF than SF (*P* = 0.019) (Fig. 1d). This difference also occurred with EMF (*P* = 0.001) and the root pathogen (*P* = 0.019), but not with mycorrhiza helper, root parasite and mildew. This difference was restored in the mycorrhiza helper-EMF interaction (*P* = 0.028). Furthermore, C allocation to sink leaves was lower during RF than SF in the control plants and with all other interactions except mildew (Fig. 1e), but again restored in the mildew-EMF treatment (*P* = 0.004). By contrast, C allocation to source leaves was higher during RF than SF (Supplementary Table S4), but only in the control (*P* = 0.002) and with EMF (*P* = 0.007). This difference was restored in EMF co-treatments with mycorrhiza helper (*P* = 0.008), root pathogen (*P* = 0.016) and mildew (*P* = 0.006). Finally, N allocation to lateral roots was higher during RF than SF only in the control (*P* = 0.014) and with EMF (*P* = 0.031; Fig. 1f). The difference in N allocation to lateral roots was not only restored, but highly significant in treatments with EMF and mycorrhiza helper (*P* < 0.001) or mildew (*P* < 0.001). At the levels of sink and source leaves as well as lateral roots, these results show that although RF/SF changes can be suppressed by interacting organisms, they are often restored in EMF co-treatments.

### Differential gene expression in oak leaves induced by a root pathogen and mildew

**Figure 2:**
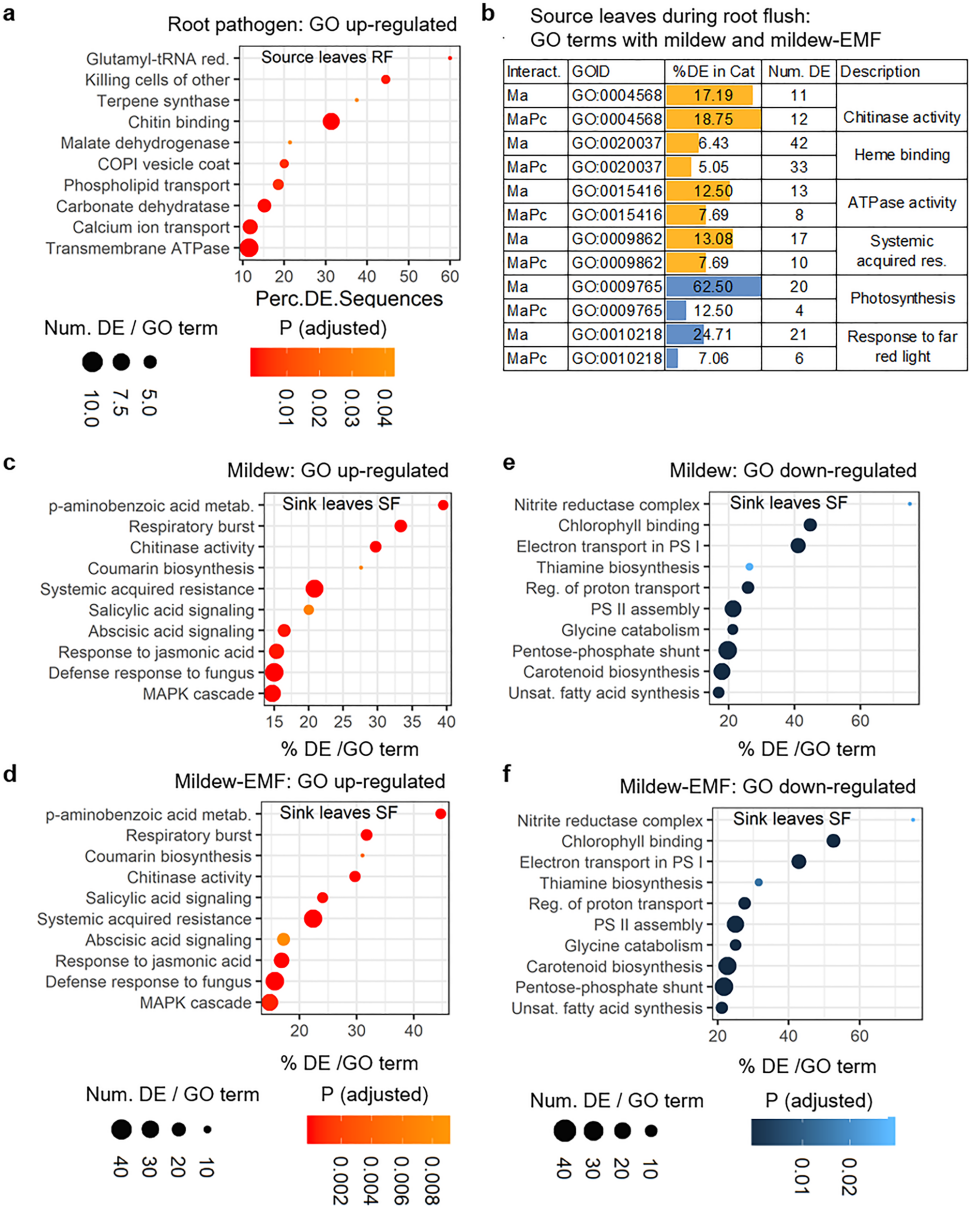
Interaction type dependent changes in the enrichment of Gene Ontology terms. Gene Ontology (GO) terms enriched in up- (orange) or down-regulated (blue) genes at the adjusted significance level of FDR < 0.05 from sink leaves during shoot flush (SF) and source leaves during root flush (RF). (**a**) Source leaves during RF with root pathogen (Pq) *Phytophthora quercina* and (**b**) with mildew (Ma) *Microsphaera alphitoides* or mildew and ectomycorrhizal fungus (EMF) *Piloderma croceum*. (**c, e**) Sink leaves during SF with mildew, and (**d, f**), with mildew and EMF. Dot plots visualise the enrichment level as the percentage of differentially expressed genes in a GO category (dot position) and as the level of significance (dot colour). Bars visualise the enrichment level as the percentage of differentially expressed sequences in a GO category (bar length).

Oak responded to the EMF, mycorrhiza helper and root parasite by differential gene expression (reviewed in^23^). To assess whether root pathogen or mildew, without and with EMF, also affect oak gene expression levels, transcript abundances in source leaves during RF and sink leaves during SF were investigated by RNA sequencing and Gene Ontology term (GO) enrichment analysis. In source leaves during RF of oaks treated with the root pathogen, 844 genes in total were differentially expressed (DEG), out of which 743 genes were up-regulated. The GO terms enriched in up-regulated genes included such related to plant defence responses, e.g. *killing cells of other organisms* and *chitin binding* (Fig. 2a).

### Biomass partitioning and resource allocation in principal roots: dependence on growth flush becomes stronger by EMF

**Figure 3:**
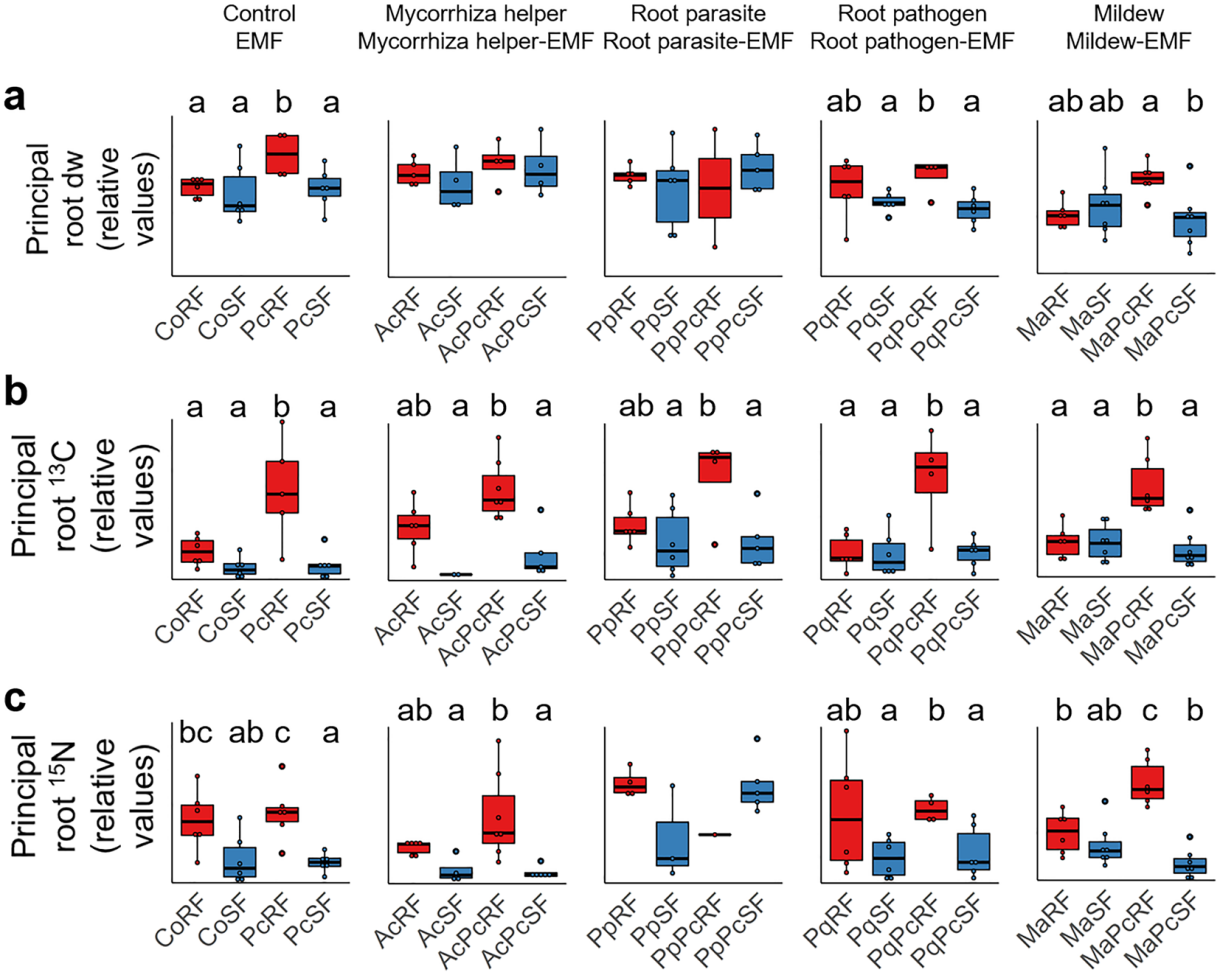
Rhythmic pattern of biomass partitioning and resource allocation is elicited by the ectomycorrhizal fungus in pedunculate oak principal roots. (**a**) Extent of biomass (g dry weight), (**b**) recently fixed carbon (excess of ^13^C), and (**c**) recently fixed nitrogen (excess of ^15^N) to the organs of oak during root and shoot flush in oak microcuttings engaged in biotic interactions. For the purpose of simplicity, relative values are shown here, and absolute values are given in Supplementary Fig. S3. Red colour marks RF and blue colour marks SF, and different letters indicate a significant difference according to linear model, ANOVA and Tukey’s test, at the significance level *P* < 0.05. Please refer to Supplementary Table S4 for the corresponding values. Where no lettering is included, the treatments were not different to each other. DW, dry weight; ^13^C, allocation of C-13; ^15^N, allocation of N-15; RF, root flush; SF, shoot flush; Pc, *Piloderma croceum*, Ac, *Streptomyces* sp. AcH505; Pp, *Pratylenchus penetrans*, Pq, *Phytophthora quercina*; Ma, *Microsphaera alphitoides.*

In non-inoculated control plants only N allocation to principal roots was higher during RF compared to SF (ANOVA and Tukey’s test; *P* = 0.025), but this changed in the presence of the EMF (Supplementary Table S4; Fig. 3). Not only N allocation to principal roots (*P* = 0.02) increased, but also C allocation (*P* = 0.004) and dry weight of principal roots (*P* = 0.03). Therefore, we asked whether the mycorrhizal fungus could increase the amplitude of RF/SF resource allocation shift at the level of the principal roots in additional interactions. The biomass of principal roots was higher during RF than during SF in EMF co-treatments with root pathogen (*P* = 0.01) and mildew (*P* = 0.027), but not in those with mycorrhiza helper or root parasite (Fig. 3a). For C allocation to principal roots (Fig. 3b) this difference was significant in all EMF co-treatments, and for N allocation to principal roots (Fig. 3c) in EMF co-treatments with mycorrhiza helper (*P* < 0.001), root pathogen (*P* = 0.042) and mildew (*P* < 0.001). These results show that plant inoculation with EMF increases the amplitude of RF/SF changes during the ERG in principal roots, and that additional interactors do not counteract this synergistic effect between the EMF and the ERG. We next addressed if the EMF restores RF/SF differences more strongly in principal than lateral roots (Supplementary Figure S3; Supplementary Table S4). For root pathogen, the RF/SF differences of biomass partitioning, C and N allocation were only significant in principal roots with additional EMF inoculation; for mildew, biomass partitioning and C allocation, and for root parasite, C allocation. By contrast, additional EMF inoculation restored the RF/SF differences of C and N allocation similarly in lateral and principal roots for helper bacterium (Supplementary Figure S3; Supplementary Table S4).

### Enhanced biomass and resource allocation during co-treatment with EMF: less RF/SF changes in stems than in other organs

**Figure 4:**
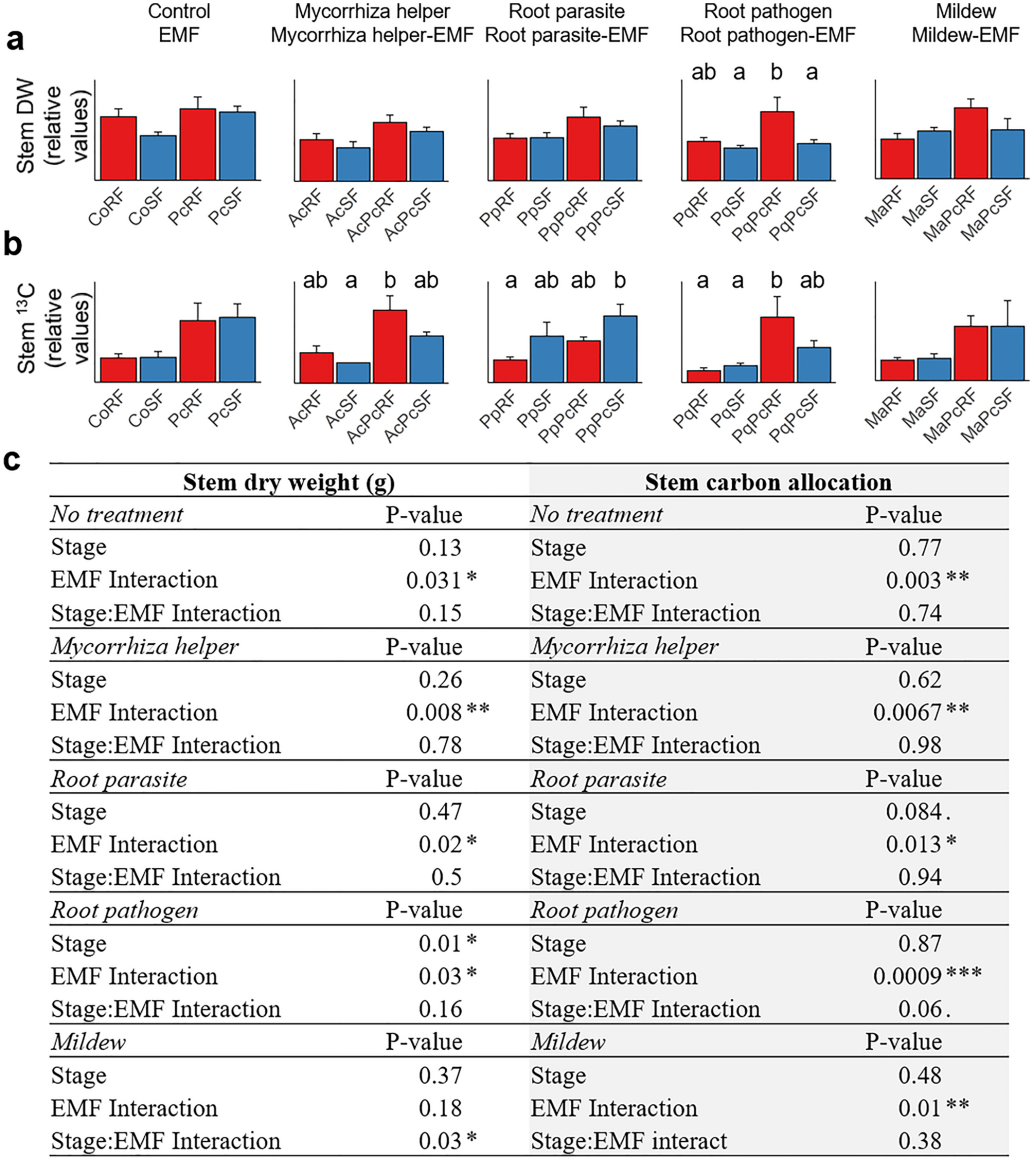
Effect of the ectomycorrhizal fungus on stem biomass and carbon allocation. (**a**) Stem biomass (g dry weight). (**b**) Carbon allocation to stem (^13^C excess). Different letters indicate a significant difference according to linear model, ANOVA and Tukey’s test, at the significance level *P* < 0.05. For the purpose of simplicity, relative values are shown here, and absolute values are given in Supplementary Fig. S3. (**c**) Effect of the EMF on biomass partitioning to stems. Data were analysed by linear model and the relative effects and the interaction between growth stage (RF/SF) and EMF (no/yes) were assessed by using two-way ANOVA. DW, dry weight; ^13^C, allocation of C-13; RF, root flush; SF, shoot flush; Pc, *Piloderma croceum*, Ac, *Streptomyces* sp. AcH505; Pp, *Pratylenchus penetrans*, Pq, *Phytophthora quercina*; Ma, *Microsphaera alphitoides.*

Co-treatments with EMF had not only an impact on leaf and root biomass and resource allocation, but also an additional positive influence on C allocation to and increased biomass of stems (Fig. 4). In contrast to other organs, the effects on stems were not related to the alternating RF/SF changes (Fig. 4a,b). EMF significantly stimulated C allocation to oak stems in all interactions (Fig. 4c) and led to increased stem biomass in co-treatments with mycorrhiza helper (ANOVA; *P* < 0.008), root parasite (*P* = 0.02) and root pathogen (﻿*P* = 0.03), but not with mildew (*P* = 0.18). Positive influence of the EMF on N allocation to stems was detected in co-treatments with mycorrhiza helper (*P* = 0.013) and root pathogen (*P* = 0.029).

Biotic interactions also modified the root-shoot ratio of C allocation (Supplementary Table S4; Supplementary Fig. S4). Whereas it was not affected by flushing in the control, it increased in RF with EMF (*P* < 0.001), with mycorrhiza helper (*P* = 0.03) and with root parasite (*P* = 0.007), and it was pronounced in EMF co-treatments with mycorrhiza helper (*P* < 0.001), root parasite (*P* < 0.004) and root pathogen (*P* < 0.001).

## Discussion

The use of pedunculate oak microcuttings that display root and shoot alternating flushing like older oak seedlings and mature trees^14^ revealed that a co-treatment with the EMF *P. croceum* can restore an altered partitioning in biomass, and C and N allocation during RF and SF induced by diverse below and aboveground interaction partners. In principal roots, this observation was even pronounced, which is remarkable, as these roots do not form ectomycorrhizas themselves. Hence, these observations implicate the following: first, a strong plant systemic effect of an EMF inoculation compared to the other interactors, and second, the existence of a fine-tuning between the EMF and the ERG in oaks.

Three forms of plant growth modulations by EMF were identified. First, the intensification of the amplitude of ERG in principal roots, which occurs at the levels of biomass and C allocation, and represents a pattern that ranges from high during RF to low during SF (Fig. 3). Second, the restoration of the expression of the ERG in oaks by the EMF, where RF/SF changes are attenuated by a detrimental biotic interaction (Fig. 1). Finally, growth promotion independently of RF and SF occurred in the intermediary connecting organ namely the stem (increased biomass and C allocation; Fig. 4c). The basic requirements for plant growth are active meristems and the availability of resources that can be allocated to these sink organs. Control of photosynthetic rate and resource allocation is exerted by the growing sink organs, i.e. the active meristems, due to resource demanding processes of tissue formation and cell growth^24^. Accounting for chemical element stoichiometry in plant tissues, the same can be implicated for N allocation^7^. During the ERG, roots and shoots alternate as strong sinks, and the ectomycorrhizal fungus increases sink strength in roots at RF and in shoots at SF^15^. Observed in stems, growth promotion independently of RF/SF changes can be explained by the importance of the phloem and xylem conduits of the stem for both roots during RF and leaves during SF, since the resources are allocated to the growing organs via the stem during both flushes.

The RF/SF changes related to the ERG were attenuated by detrimental interactions including the root parasite and root pathogen that colonize the roots and the mildew that infects the leaves. The observations confirm our first hypothesis stating that plant response to detrimental organisms alters the basic RF/SF allocation pattern. RF/SF changes were also in part suppressed by mycorrhiza helper, and this response of the oak may be explained by the earlier gene expression response data. Although mycorrhiza helper stimulates mycorrhiza formation, it causes a systemic stress response in oak when inoculated in the absence of the EMF^21^. Maximal C allocation to oak roots during RF stimulates the infection of roots by the root parasite^19^, similar to the root pathogen^25^. N allocation to lateral roots was not higher during RF than SF with root parasite, and principal root dry weight and N allocation were not higher during RF with root parasite or root parasite-EMF. This suggests that higher rate of parasitism in RF roots^25^ causes attenuation of RF/SF changes. Together, these observations support our second hypothesis that the presence of EMF partly restored the RF/SF changes, when the EMF was combined with interactions that alone attenuated the RF/SF changes. Moreover, in root parasite colonised plants, neither the extent of biomass nor the C allocation to source leaves were higher in RF than in SF. This suggests that the infection by the root parasite has a systemic effect on resource allocation during RF. At the gene expression level, the presence of systemic responses was supported by oak leaf RNA sequencing analysis^22^ that suggested changes at the levels of cell growth and C metabolism, as well as a plant defence response. Consistent with the current observations, infection by the root parasite functions as a sink for resources in radiate pine, reduces root and shoot biomass, decreases carbohydrate reserves and inhibits ectomycorrhiza development^26^. Data from potato show that the root parasite infection does not significantly limit photosynthesis^27^, suggesting that the negative impact is associated with increased sink strength.

Our third hypothesis stated, that the restoration of RF/SF biomass partitioning and resource allocation by the EMF are expressed particularly strongly in principal roots. The data (Supplementary Figure S3; Supplementary Table S4) support, but only in part, this hypothesis, since whereas EMF based restoration was evident only in principal roots for root pathogen, it had a similar effect on RF/SF differences of C and N allocation in lateral and principal roots for helper bacterium. This suggests that further factors, perhaps related to the direct interaction between the EMF and the helper bacterium, such as biotic stress experienced by the EMF^28,29^, may modulate the restoration of RF/SF biomass partitioning and resource allocation by the EMF.

**Figure 5:**
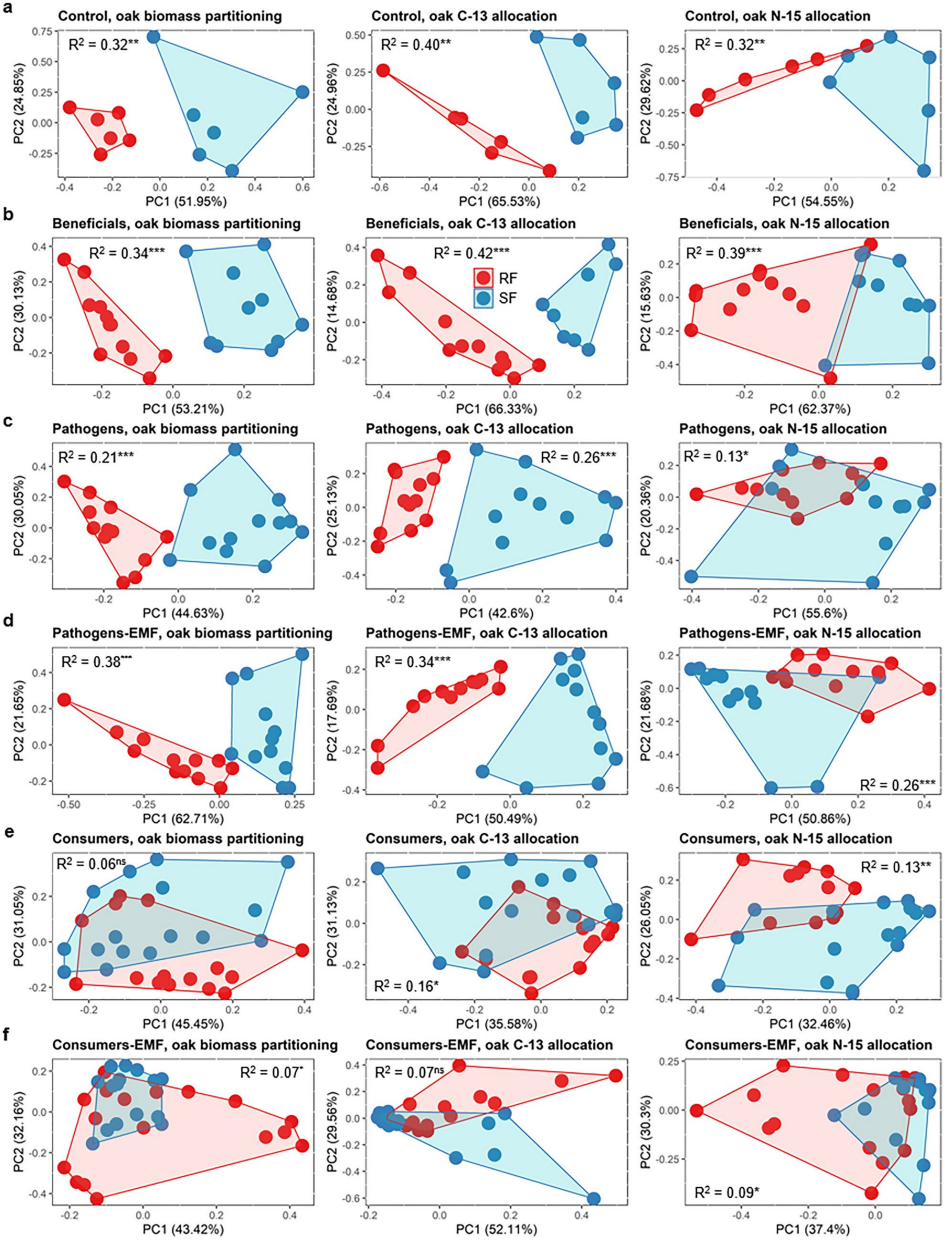
Growth stage dependent distribution of biomass and resource allocation in oak: Summary. Principal components analysis of the combined variables of sink and source leaves, stems, principal and lateral roots: biomass (dry weight), carbon allocation (^13^C excess) and nitrogen allocation (^15^N excess). Treatments comprise (**a**) control treatment, (**b**) EMF and mycorrhiza helper (beneficials), (**c**) root pathogen and mildew (pathogens), and with additional EMF (**d**), (**e**) root parasite, springtail and leaf herbivore (consumers), and with additional EMF (**f**). Springtail (*Protaphorura armata*) and Springtail-EMF data were obtained from Graf et al. (2019) and leaf herbivore (*Lymantria dispar*) and leaf herbivore-EMF data from Bacht et al. (2019). Each symbol represents the total distribution of biomass or resource allocation in an oak individual. Data points for each group are enclosed with a line, and red colour marks RF and blue colour marks SF. Based on a permutational multivariate analysis of variance, R-squared (R^2^) represents the proportion of the variance that's explained by growth stage, and asterisks indicate significant differences between the growth stages at the significance levels ***(*P* < 0.001); **(*P* < 0.01); *(*P* < 0.05); ns (not significant).

The data on oak biomass partitioning and resource allocation presented here and in previous papers are summed up in an interaction type related manner in Fig. 5. This includes interactions with the leaf herbivore *Lymantria dispar* and the root interacting springtail *Protaphorura armata*^17,18^. Data from oak beneficial organisms, i.e. the EMF and the helper bacterium, did not affect the RF-SF pattern (Fig. 5a,b). Pathogens, root pathogen and leaf mildew altered the RF-SF pattern of nitrogen allocation (Fig. 5c), but this was negated in the EMF co-treatments (Fig. 5d). By contrast, the RF-SF pattern was strongly altered by consumers (root parasite, springtail and root herbivore) (Fig. 5e), but not restored by the EMF (Fig. 5f). This may have been due to three alternative mechanisms: the interactions with the consumers affect (1) the growth and physiology of the EMF mycelium, (2) the interaction between the EMF and the oak, and/or (3) the growth and physiology of the oak. Presumably, these factors acted in concert. Colonisation of roots by *Pratylenchus* spp. inhibits EM formation of forest trees^26,30^, causes a reduction of the rhizosphere microbial biomass^31^ and a reduction in root growth^32^. The springtail *P. armata* can acquire nutrients from litter, but in presence of plant roots they may switch diet and obtain both C and N almost exclusively from plant roots^33^.

Collembola can also feed on EMF mycelium and inhibit EM formation^34^, but since the grazing may stimulate compensatory growth of the hyphae, mycelial biomass may not decrease but even increase due to moderate grazing^29,35^. Finally, some reports show that leaf herbivory inhibits EM formation^36,37^, but it may also speed up assimilate transport to roots^18,38^. In sum, this suggests that consumers cause changes in root growth and resource allocation, and affect the mycelium of the EMF and its interactions with fine roots. By these mechanisms, they modulate the RF/SF changes, also in presence of EMF.

Previous analyses of oak gene expression had been conducted with mycorrhiza helper^21^, root parasite^22^, springtail^17^, and leaf herbivore^18^. These analyses showed strong gene expression responses with > 1000 differentially expressed genes, with enriched GO terms in up-regulated genes for plant defence and stress, but enriched GO terms in down-regulated genes for depleted cell growth and cell proliferation. The gene expression responses in these earlier analyses were attenuated by an additional EMF treatment—an indication of lower stress and increased potential for growth in co-treated oaks (see also^23^). In the present report, root pathogen induced a moderate response of 844 differentially expressed genes with up-regulated genes for e.g., defense response and terpenoid synthesis, the number dropped to 127 differentially expressed genes with EMF co-treatment, which is in line with previous observations and our fifth hypothesis^18,21,22^. Unexpectedly, mildew and mildew-EMF treatments altered the expression of a similar number of differentially expressed genes, and induced or repressed functionally similar sets of genes, in sink leaves during SF and in source leaves during RF. This suggests that the restoration of RF/SF changes by EMF does not necessarily involve extensive modifications in oak gene expression levels, and that post-transcriptional and post-translational processes play their respective roles in this restoration.

Studies analyzing modifications of plant growth in response to EMF often found an increase in total plant biomass, and in particular, enhanced formation of lateral roots^39^. During this process, the ectomycorrhizal fungi engage in a molecular dialogue with plants. Lateral root formation is stimulated by EMF producing auxins, ethylene and/or sesquiterpenes (reviewed in^40^). The existing palette of beneficial traits of the EMF (reviewed in^41^) was expanded by showing that the EMF restored the RF/SF changes that were attenuated in particular by detrimental biotic interactions, and intensified RF/SF changes in principal roots.

Our study raises a new question how EMF supports RF/SF changes in oaks, and what are the fungal and plant communication pathways behind this beneficial impact of the symbiont on ERG? Our results provide a limited snapshot, and biotic interactions during oak development may be undergoing changes. For instance, Maboreke et al*.*^31^ showed that the effect of springtails and nematodes on the rhizosphere microbial biomass increased and their impact on microbial community composition varied with time, suggesting that the age of oak microcuttings is an important additional driver of biotic interactions. Finally, ecological data from multi-trophic interactions and experiments modulating abiotic and biotic factors will be keys in assessing the relevance of EMF-based stimulation or parasite-based inhibition of rhythmic growth pattern in oaks and other rhythmic growing trees.

## Materials and methods

### Plant cultivation and biotic interactions

The pedunculate oak (*Q. robur*) clone DF159 was micro-propagated and rooted according to^42^. Microcuttings were cultivated in a 12 × 12 cm^2^ Petri dish system using gamma sterilized soil in the root compartment and placing shoots outside the Petri dish as presented by Herrmann et al*.*^15,23^. During establishment of the system, half of the microcuttings was inoculated with the ectomycorrhizal fungus *P. croceum* (strain F1598,^23^), and the other half was used for non-inoculated controls. After three weeks cultivation, 5 ml of filtrate of the same forest soil that was used as a substrate after gamma sterilization for experimentation was added to each culture system as described by Rosenberg et al*.*^43^ to establish a natural microbial community and thereby making the system closer to natural conditions. The ectomycorrhizal fungus treatment was combined in a factorial design with inoculation of one of six different biotrophic partners that were introduced later to the culture system (Supplementary Table S1; see^23^ for more details). We tested the single interaction and the co-treatment with the mycorrhizal fungus for each interactor (no treatment, interactor, EMF, interactor-EMF). Briefly, mycorrhiza helper *Streptomyces* sp. AcH 505^44^ was inoculated on oak roots twice (3 weeks and 1.5 weeks before harvesting) with 2.5 × 10^7^ bacterial spores each (see^21^ for details). The root parasitic nematodes *Pratylenchus penetrans* (Cobb) Philip & Stek were kindly provided by Johannes Hallman (Julius Kühn-Institute, Germany) and applied on the root system at the number of ≈10,000 nematodes 7 days before harvesting (see^22^ for details). The root pathogen *P. quercina* T. Jung strain QUE 6 (CBS 789.95) was inoculated on oak root system using 1 × 10^6^ zoospores per plant one week before harvesting (see^25^ for details). The powdery mildew *Microsphaera alphitoides* was obtained from mildew-infected oak trees and maintained on oak, before inoculating oak buds and young leaves of the DF159 microcuttings by using 1.5 × 10^6^ mildew spores per plant two weeks before harvesting (see^20^ for details). The development of the plants was monitored in growth chambers providing 23 ± 1 °C and long-day (16 h light/8 h dark) conditions with a photosynthetic photon flux density of ~ 180 μmol m^−2^ s^−1^ and 75% relative air humidity. Optimal growth conditions of 23 °C and long days are required for the expression of the endogenous rhythmic growth of pedunculate oaks^9,14^. Under these conditions the flushing period is about 4 to 5 weeks^9,14^. Each microcosm was watered with sterilized tap water every 14 d using a sterile syringe in order to maintain the original weight of the microcosm. The developmental stage and numbers of established shoot flushes were recorded biweekly in a non-destructive manner. Four developmental stages characterize each growth cycle of the oak: the bud rest stage A, the bud swelling stage B that tightly correlates with maximal root elongation rate, the shoot elongation stage C, and the leaf expansion stage D (^14^; Supplementary Fig. S1). Under the used experimental conditions, the first ectomycorrhizas formed during the fifth week. Even so, only few mycorrhizas were detected at harvest time, 11 weeks after the inoculation with *P. croceum*. This was expected, since growth conditions included a relatively high air humidity and sufficient water supply to minimize plant stress during cultivation, which also reduce the differentiation of EMF^15^. Harvest was performed at developmental stage B for RF and developmental stage D for SF. The harvested shoot tissues consisted of sink buds in RF and sink leaves in SF, source leaves and stems, and the root tissues from lateral and principal roots (see^23^ for details). Of note, even under optimal growth conditions, the oak microcuttings are not synchronous in their growth pattern. This enabled us to sample the oaks during RF and during SF at the total age of 11 weeks. After harvest, the tissues were immediately submerged in liquid nitrogen. For resource allocation analyses, each component was dried to a constant mass, milled to homogeneity and analysed for its C and N stable isotope composition on an isotope ratio mass spectrometer (Isoprime; Elementar, Hanau, Germany; for details see^23^). The total number seedlings was 120.

### Labelling and estimation of recently assimilated carbon and nitrogen

The allocation patterns of C and N were assessed by stable isotope labelling. Three days before harvesting, 5 ml of 0.02 g l^−1^, ^15^NH_4_^15^NO_3_ (98 atm% ^15^N, Sigma, Germany) were added to each root compartment under sterile conditions. For ^13^CO_2_ labelling, microcosms were transferred into a Plexiglas chamber 36 h before harvest. During the night before labelling, the CO_2_ in the atmosphere was scrubbed with soda lime and replaced with CO_2_ with 10 atm% ^13^CO_2_ (Eurisotop, Saarbrücken, Germany). During the following daytime period (16 h), the CO_2_ concentration was adjusted to 400 ± 2 μl l^−1^ (mean ± SD) with a ^13^C atm% of 7.9 ± 0.3 (mean ± SD). Carbon isotopic composition of atmospheric samples was assessed using an isotope ratio mass spectrometer (Isoprime; Elementar, Hanau, Germany). For further details see^23^. In the used culture system, we expect that the ^13^C and ^15^N excess values result from the assimilation by the plant. The ^13^CO_2_ is assimilated by the plant, and none of the interacting organisms can fix atmospheric ^13^CO_2_. The ^15^N is taken up by the roots, and not by the ectomycorrhizal fungus. This is, because in the used culture system only few ectomycorrhizas formed (< 1% of fine root biomass). Thus, we interpret changes in N and C allocation as an indication of plant response to the interacting organism.

### RNA sequencing

Total RNA was extracted using the MasterPure Plant RNA Purification Kit (Epicentre, Hessisch Oldendorf, Germany) with 100 mg of root or 50 mg of leaf material per extraction. RNA quality and quantity were verified using a NanoDrop 1000 spectrophotometer and an Agilent 2100 Bioanalyzer. One hundred bp paired-end Illumina Truseq version 2 libraries were constructed, quality checked, and sequenced at the average depth of 15 million reads per sample using the Illumina HiSeq2000 sequencing platform at the Beijing Genomics Institute (Hong Kong, China). The raw sequences are available as fastq files at the NCBI Short Read Archive, linked to BioProject accessions termed PRJNA702914 (root pathogen, root pathogen-EMF) and PRJNA702917 (mildew, mildew-EMF). Reads were processed following the protocol in Tarkka et al*.*^45^. Briefly, low quality sequences and sequencing artefacts were removed with SeqClean (http://sourceforge.net/projects/seqclean/files/) and low quality sequencing ends were trimmed with a custom Java script. Short sequences (< 50 bp) and sequences lacking paired-end information were discarded. The processed Illumina reads were aligned against the reference transcriptome OakContigDF159.1^45^ by Bowtie^46^. Mapping rates to the reference transcriptome varied between 76 and 83%. The relative gene expression levels were quantified by RSEM^47^ and fold changes in gene expression were calculated by pairwise comparisons using the edgeR function^48^ implemented in the Bioconductor package^49^. Genes were called to be differentially expressed in a treatment compared with the corresponding control condition when exhibiting a Benjamini–Hochberg adjusted *P* < 0.01 after adjustment for multiple testing. Resulting gene lists were analysed for enriched GO terms and KEGG pathways using the Bioconductor package GOseq^50^. The OakContigDF159.1 reference library, GO annotations as well as best blast hits of each contig have been deposited at www.trophinoak.de. The reliability of the RNA-Seq analyses has been demonstrated earlier by Real-time-quantitative reverse transcriptase-PCR^15,20,21^.

### Statistics

All statistical analyses were performed in the open source program R version 3.6.2 (2019–12-12), GNU project (R Core Team 2019). Permutational multivariate analysis of variance (PERMANOVA) was used to test for RF/SF differences in the relative distributions of dry weight, carbon and nitrogen allocation in the oak microcuttings. Datasets were tested for normality and skewed data were log-transformed. Dry weight, C and N allocation at the level of individual plant compartments were analysed by linear model with the lm function in R and subjected to the analysis of variance and multiple comparison analysis with Tukey’s HSD tests. Significance levels were defined as *p* < 0.001***, *p* < 0.01** and *p* < 0.05*. The number of replicates of the individual variables differed between the treatments due to in part uneven numbers of plants in SF and RF, and the replicate numbers are given in Supplementary Table S2. Whereas most samples included 6 replicates, for the analysis of C allocation for mycorrhiza helper in SF, the replicate number was 2. Additionally to Supplementary Table S2, this case is reported in the legends of Figs. 1, 2 and 4.

## Supplementary Information

Supplementary Information.

Supplementary Table S4.

Supplementary Legends.

## Data Availability

Biomass partitioning, carbon and nitrogen data supporting the findings of this study are available within the paper and within its supplementary materials published online. The transcriptome sequencing data is available at the NCBI under the Bioprojects PRJNA702914 (root pathogen, root pathogen-EMF) and PRJNA702917 (mildew, mildew-EMF). *Quercus robur* clone DF159 plants were used in this study. The oak genotype DF159 corresponds to a seedling of the French "paradoxal" oak 159. Prof. Dr. J.-M. Favre (University of Nancy, France) kindly provided its clone in 1990. We have the permission to carry out experiments with the oak clone DF159. We implemented experimental research and harvests of *Quercus robur* clone DF159 plants according to the institutional, national, and international guidelines and legislation.
