# Supplementary material for: Ectomycorrhizal fungus supports endogenous rhythmic growth and corresponding resource allocation in oak during various below- and aboveground biotic interactions
Source: Sci Rep. 2021 Dec 8;11:23680. doi: 10.1038/s41598-021-03132-y (PMC8654951; doi:10.1038/s41598-021-03132-y)

Supplementary Table S1. Treatments of pedunculate DF159 oak (*Quercus robur*) microcuttings with five interacting organisms. Day 0 indicates the date on which oak microcuttings were placed in soil microcosms. After 3 weeks, soil filtrate was added to each culture system to establish a natural microbial community. The microcuttings were grown in Petri dish soil microcosms in total for 11 weeks to produce the plant material for the analysis. For co-treatments with the ectomycorrhizal fungus (EMF), the plants were inoculated at day 0 with the fungus in the same manner as in single EMF treatment.

**Sample**	**Treatment type**	**Description of the treatment**	**Timing before harvest**
No treatment	No inoculation	No inoculation	
EMF	*Piloderma croceum*	Fungal inoculum was mixed with the soil substrate once, at day 0	11 wk
EM helper bacterium	*Streptomyces* sp. AcH 505	2.5×10^7 spores were applied to the roots twice	3 & 1.5 wk
Leaf pathogen	*Microsphaera alphitoides*	1.5×10^6 spores were applied to leaves once	2 wk
Root pathogen	*Phytophthora quercina*	1.0×10^6 zoospores per plant were applied to the roots once	1 wk
Root parasite	*Pratylenchus penetrans*	$\approx 1.0 \times 10^4$ nematodes per plant were applied to the roots once	1 wk

Supplementary Table S2. Numbers of samples obtained for the estimation of the distribution of (A) biomass, (B) carbon and (C) nitrogen allocation during biotic interactions. The sample numbers <5 are highlighted as bold letters. Pc = EMF *Piloderma croceum*, Ac = mycorrhiza-helper *Streptomyces* sp. AcH 505; Pp = root parasite *Pratylenchus penetrans*, Pq = root pathogen *Phytophthora quercina*; Ma = mildew *Microsphaera alphitoides.* The corresponding values for principal roots with *Protaphorura armata* were during RF 4 and SF 4, and for *P. armata*-EMF interaction during RF 6 and SF 5 samples, and for principal roots with *Lymantria dispar* and *L. dispar*-EMF six samples for RF and SF, respectively.

(A) Biomass (dry weight) sample number

Treatment	DWsink	DWsource	DWstem	DWLR	DWPR
CoRF	6	6	6	6	6
CoSF	6	6	6	6	6
PcRF	6	6	6	6	6
PcSF	6	6	6	6	6
AcRF	6	6	6	6	6
AcSF	**4**	**4**	**4**	**4**	**4**
AcPcRF	7	7	7	7	7
AcPcSF	6	6	6	6	6
PpRF	6	6	6	6	6
PpSF	6	6	6	6	6
PpPcRF	6	6	6	6	6
PpPcSF	5	5	5	5	5
PqRF	6	6	6	6	6
PqSF	6	6	6	6	6
PqPcRF	6	6	6	6	6
PqPcSF	6	6	6	6	6
MaRF	6	6	6	6	6
MaSF	7	7	7	7	7
MaPcRF	6	6	6	6	6
MaPcSF	7	7	7	7	7

(B) C-13 allocation sample number

Treatment	13Csink	13Csource	13Cstem	13CLR	13CPR
CoRF	6	6	6	6	6
CoSF	6	6	6	6	6
PcRF	6	6	6	6	6
PcSF	6	6	6	6	6
AcRF	6	6	6	6	6
AcSF	**2**	**2**	**2**	**2**	**2**
AcPcRF	7	7	7	7	7
AcPcSF	5	5	5	5	5
PpRF	6	6	6	6	6
PpSF	6	6	6	6	6
PpPcRF	6	6	6	6	6
PpPcSF	5	5	5	5	5
PqRF	6	6	6	6	6
PqSF	6	6	6	6	6
PqPcRF	6	6	6	6	6
PqPcSF	6	6	6	6	6
MaRF	6	6	6	6	6
MaSF	7	7	7	7	7
MaPcRF	6	6	6	6	6
MaPcSF	7	7	7	7	7

(C) N-15 allocation sample number

Treatment	15Nsink	15Nsource	15Nstem	15NLR	15NPR
CoRF	6	6	6	6	6
CoSF	6	6	6	6	6
PcRF	6	6	6	6	6
PcSF	6	6	6	6	6
AcRF	6	6	6	6	6
AcSF	**4**	**4**	**4**	**4**	**4**
AcPcRF	7	7	7	7	7
AcPcSF	6	6	6	6	6
PpRF	8	8	8	8	8
PpSF	6	6	6	6	6
PpPcRF	6	6	6	6	6
PpPcSF	5	5	5	5	5
PqRF	6	6	6	6	6
PqSF	6	6	6	6	6
PqPcRF	6	6	6	6	6
PqPcSF	6	6	6	6	6
MaRF	6	6	6	6	6
MaSF	7	7	7	7	7
MaPcRF	6	6	6	6	6
MaPcSF	7	7	7	7	7

Supplementary Table S3. Effect of growth stage on the distribution of biomass, and carbon and nitrogen allocation in oak during biotic interactions, PERMANOVA analysis. Distribution of biomass, recently assimilated carbon and nitrogen in sink and source leaves, stem, lateral and principal roots of oak microcuttings at root flush and shoot flush were compared. Permutational multivariate analysis of variance (PERMANOVA) on Euclidean distances on resource allocation parameters according to growth stages B and D of oak, root flush and shoot flush, respectively. N, number of oak microcuttings; SS, sum of squares; F.Model, F value by permutation; R^2, effect size; P(perm.), P-value. Asterisks indicate significant differences at the levels of * ($P<0.05$), ** ($P<0.01$), *** ($P<0.001$). DW, dry weight; C-13, allocation of C-13; N-15, allocation of N-15; RF = root flush; SF = shoot flush; Pc = *Piloderma croceum*, Ac = *Streptomyces* sp. AcH 505; Pp = *Pratylenchus penetrans*; Pq = *Phytophthora quercina*; Ma = *Microsphaera alphitoides*. Note that for AcSF C-13 n = 2

Interaction and trait	N	SS	F-Model	R^2	P(perm.)	
Control, oak DW	12	17.6	47	0.31961	0.01	**
Control, oak C-13	12	22.2	67.6	0.40335	0.009	**
Control, oak N-15	12	17.7	47.4	0.32156	0.006	**
Pc, oak DW	12	22.3	68.4	0.40617	0.002	**
Pc, oak C-13	12	24.2	78.4	0.43953	0.004	**
Pc, oak N-15	12	21.2	62.9	0.3863	0.008	**
Ac, oak DW	10	13.4	34.1	0.2988	0.034	*
Ac, oak C-13	8	15.8	49.2	0.45069	0.076	.
Ac, oak N-15	10	22.2	7.8	0.49235	0.019	*
AcPc, oak DW	13	19.3	52.3	0.32206	0.001	***
AcPc, oak C-13	12	27.5	10	0.52196	0.01	**
AcPc, oak N-15	13	40.1	22.1	0.66754	0.002	**
Pp, oak DW	12	8.475	18.216	0.15409	0.117	
Pp, oak C-13	12	10.614	23.912	0.1928	0.052	.
Pp, oak N-15	12	8.889	19.278	0.16162	0.072	.
PpPc, oak DW	11	9.941	22.333	0.19881	0.057	.
PpPc, oak C-13	11	13.869	34.546	0.27738	0.005	**
PpPc, oak N-15	11	10.403	23.645	0.20806	0.029	*
Pq, oak DW	12	17.4	46.3	0.31666	0.009	**
Pq, oak C-13	12	17.3	46.1	0.31532	0.002	**
Pq, oak N-15	12	17.6	47.2	0.32045	0.001	***
PqPc, oak DW	12	28.7	10.9	0.31961	0.008	**
PqPc, oak C-13	12	21.1	62.1	0.39998	0.005	**
PqPc, oak N-15	12	13.2	31.5	0.32156	0.01	**
Ma, oak DW	13	16.7	42.4	0.27822	0.004	**
Ma, oak C-13	13	14.1	33.7	0.23454	0.005	**
Ma, oak N-15	13	3.8	0.7	0.06255	0.536	
MaPc, oak DW	13	19.8	54.1	0.32953	0.007	**
MaPc, oak C-13	13	25.3	79.9	0.4209	0.002	**
MaPc, oak N-15	13	26.6	87.4	0.44288	0.001	***

Supplementary Table S4. Effects of developmental stage and additional treatment with mycorrhizal fungus on the distribution of plant biomass, carbon and nitrogen allocation at the level of plant organs. Data were analysed by linear model, two-way ANOVA (the relative effects and the interaction between growth stage (RF/SF) and EMF (no/yes)), and Tukey HSD test. First sheet of the data concerns the datasets from the plant compartments and second sheet from the root/shoot ratios. For sample numbers see Table S2. This table represents the statistics used to distinguish the differences between the treatments. DW, dry weight; C-13, allocation of C-13; N-15, allocation of N-15; RF = root flush; SF = shoot flush; Pc = *Piloderma croceum*, Ac = *Streptomyces* sp. AcH 505; Pp = *Pratylenchus penetrans*; Pq = *Phytophthora quercina*; Ma = *Microsphaera alphitoides*. Note that for AcSF C-13 sink n = 2. File Table S4.xlsx.

Supplementary Figure S1. Schematic representation of the developmental stages during root flush and shoot flush in oak microcuttings. Arrows mark the sink and the source leaves.

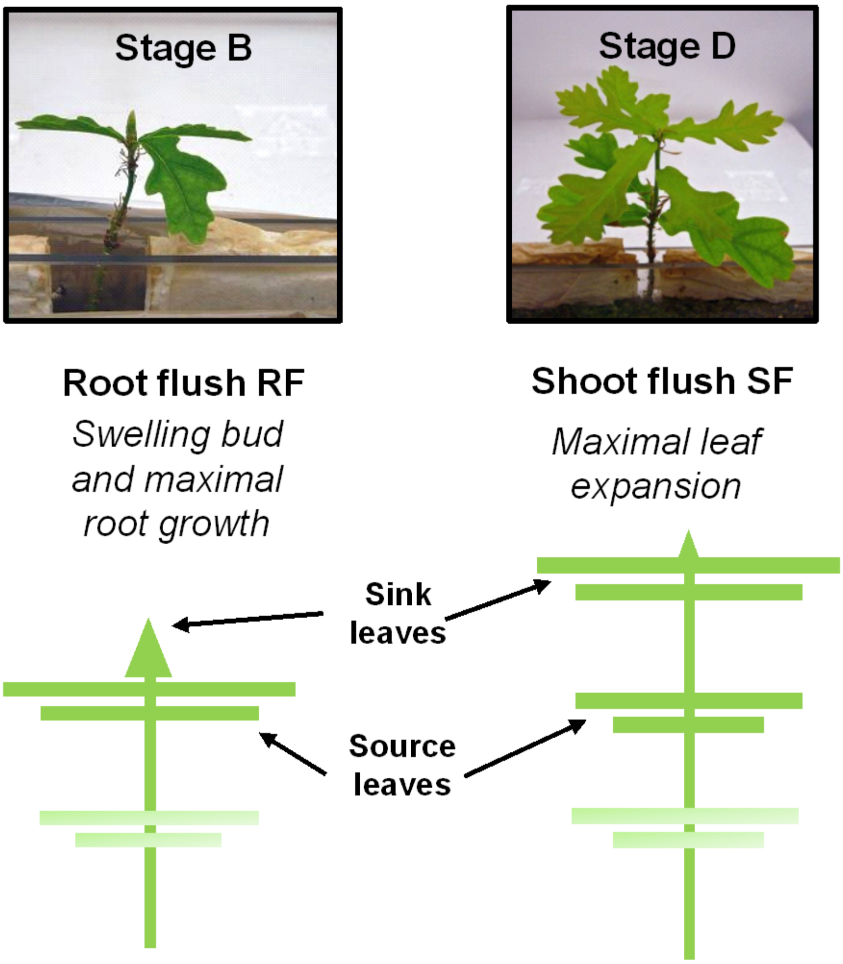

Supplementary Figure S2. Principal components analysis of the distribution of biomass, and the allocation of recently fixed carbon and nitrogen to the sink and source leaves, stems, primary and lateral roots of oak microcuttings. Each symbol represents the total distribution of resources in an oak individual. Data points for each group are enclosed with a line, and red colour marks RF and blue colour marks SF. Stars indicate difference according to permutational multivariate analysis of variance (PERMANOVA) on Euclidean distances on resource allocation, at the significance levels * ($P<0.05$), ** ($P<0.01$), *** ($P<0.001$). Please consult Table S3 for the PERMANOVA values. DW, dry weight; 13C, allocation of C-13; 15N, allocation of N-15; RF = root flush; SF = shoot flush; Pc = *Piloderma croceum*, Ac = *Streptomyces* sp. AcH 505; Pp = *Pratylenchus penetrans*; Pq = *Phytophthora quercina*. Note that the corresponding Fig.s of the treatment with mildew are shown in Fig.s 1a and 1b.

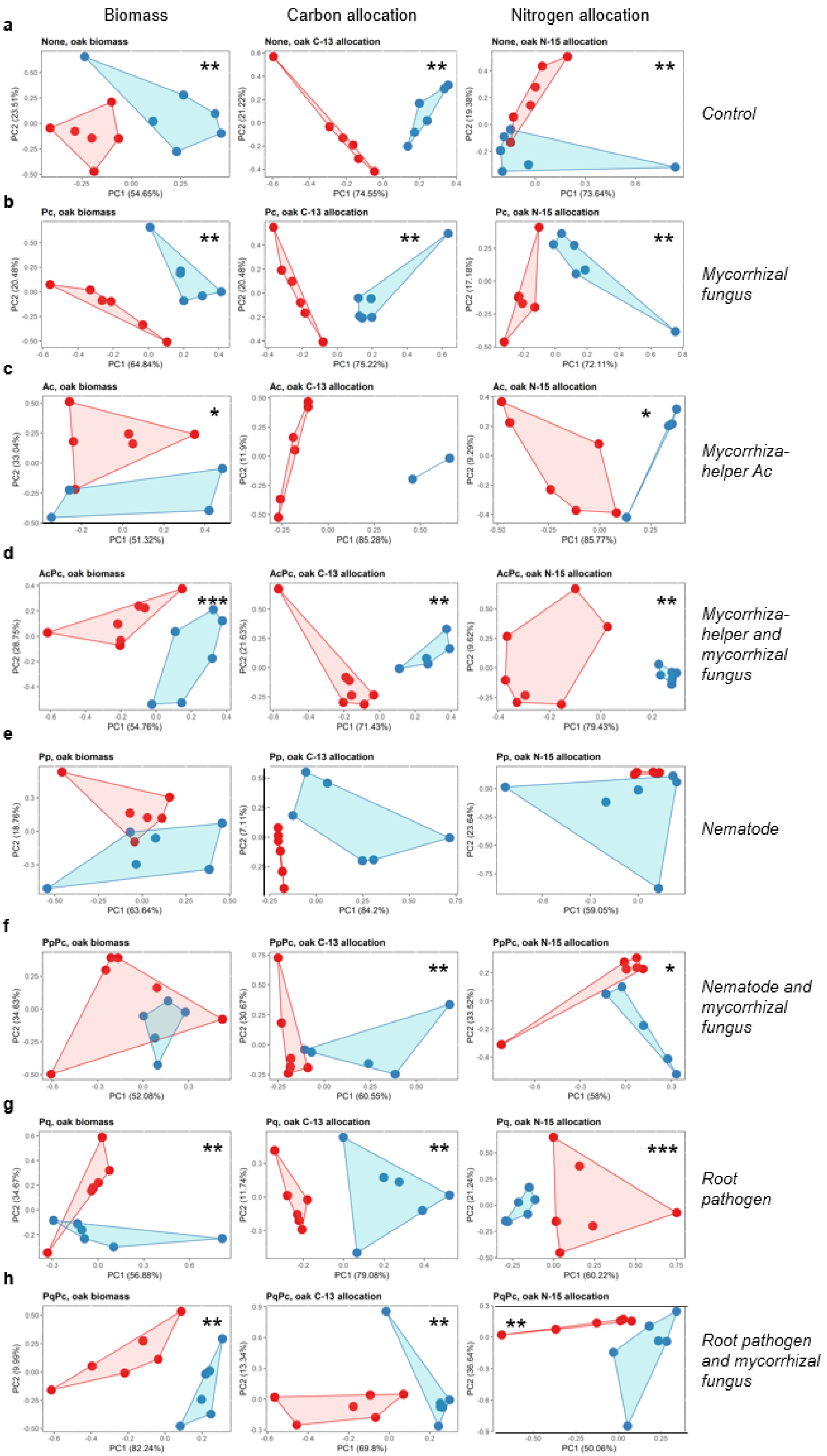

a
b
c
d
e
f
g
h
Biomass
Carbon allocation
Nitrogen allocation
None, oak biomass
None, oak C-13 allocation
None, oak N-15 allocation
Control
Pc, oak biomass
Pc, oak C-13 allocation
Pc, oak N-15 allocation
Mycorrhizal fungus
Ac, oak biomass
Ac, oak C-13 allocation
Ac, oak N-15 allocation
Mycorrhiza-helper Ac
AcPc, oak biomass
AcPc, oak C-13 allocation
AcPc, oak N-15 allocation
Mycorrhiza-helper and mycorrhizal fungus
Pp, oak biomass
Pp, oak C-13 allocation
Pp, oak N-15 allocation
Nematode
PpPc, oak biomass
PpPc, oak C-13 allocation
PpPc, oak N-15 allocation
Nematode and mycorrhizal fungus
Pq, oak biomass
Pq, oak C-13 allocation
Pq, oak N-15 allocation
Root pathogen
PqPc, oak biomass
PqPc, oak C-13 allocation
PqPc, oak N-15 allocation
Root pathogen and mycorrhizal fungus

Supplementary Figure S3. Extent of biomass, recently fixed carbon and nitrogen in the individual organs of oak during root and shoot flush in oak microcuttings engaged in biotic interactions. Resource levels in sink and source leaves, stems, primary and lateral roots are shown by boxplots. The boxplots include a line marking the median, middle box representing the middle 50% of scores for each treatment, as well as whiskers and dots representing the scores outside and strongly deviating from the middle 50% of the scores. Different letters indicate a significant difference ($P<0.05$) based on an analysis using a linear model, ANOVA and Tukey test. Please refer to Table3 for the values of difference. Where no lettering is included, the treatments were not different to each other. DW, dry weight; 13C, allocation of C-13; 15N, allocation of N-15; RF = root flush; SF = shoot flush; Pc = *Piloderma croceum*, Ac = *Streptomyces* sp. AcH 505; Pp = *Pratylenchus penetrans*; Pq = *Phytophthora quercina*; Ma = *Microsphaera alphitoides*. Please refer to Table S2 for the numbers of replicates and note that for AcSF C-13 samples n = 2.

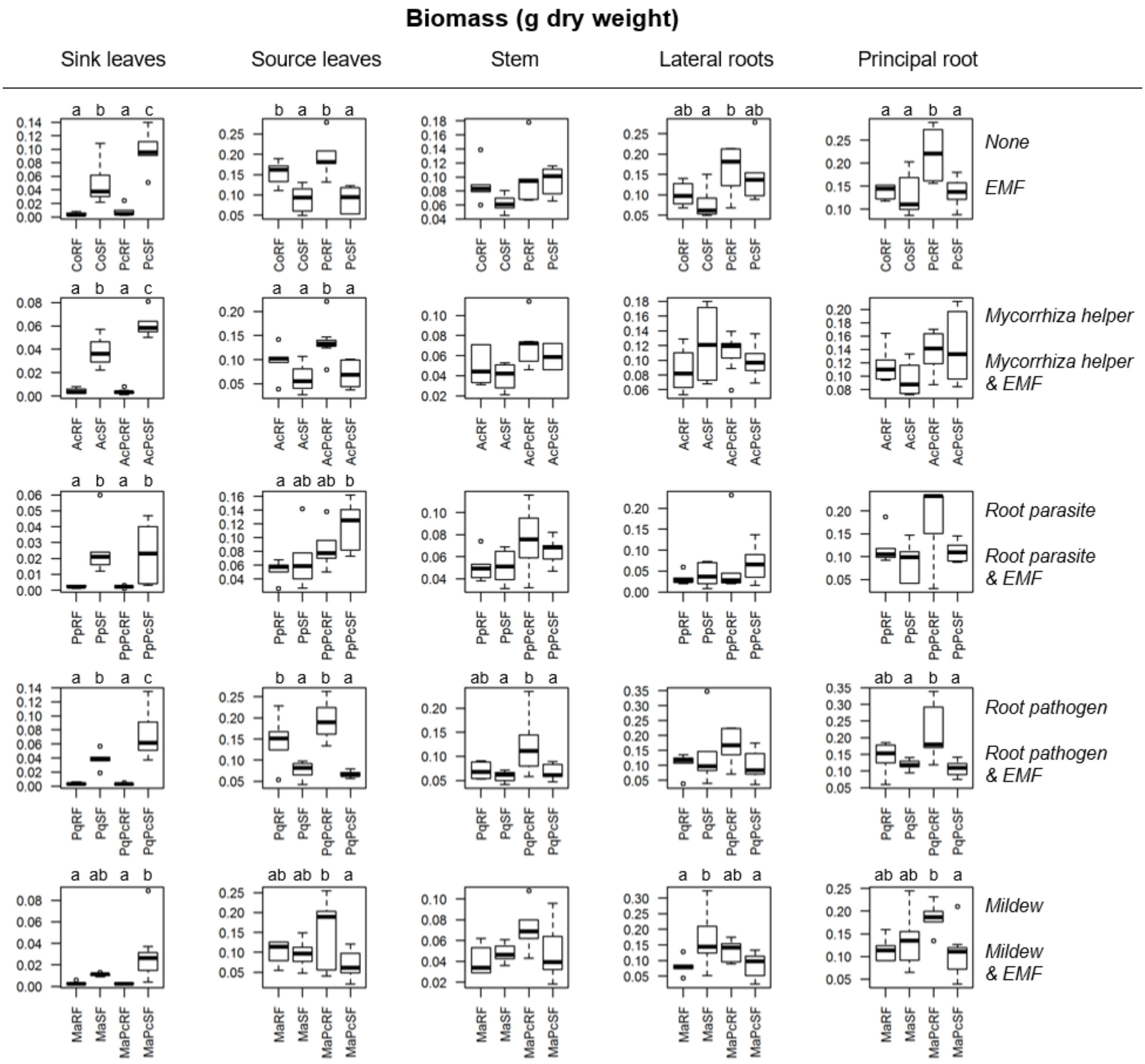
Biomass (g dry weight)
Sink leaves
Source leaves
Stem
Lateral roots
Principal root
None
EMF
Mycorrhiza helper
Mycorrhiza helper & EMF
Root parasite
Root parasite & EMF
Root pathogen
Root pathogen & EMF
Mildew
Mildew & EMF
CoRF CoSF PcRF PcSF
AcRF AcSF AcPcRF AcPcSF
PpRF PpSF PpPcRF PpPcSF
PqRF PqSF PqPcRF PqPcSF
MaRF MaSF MaPcRF MaPcSF

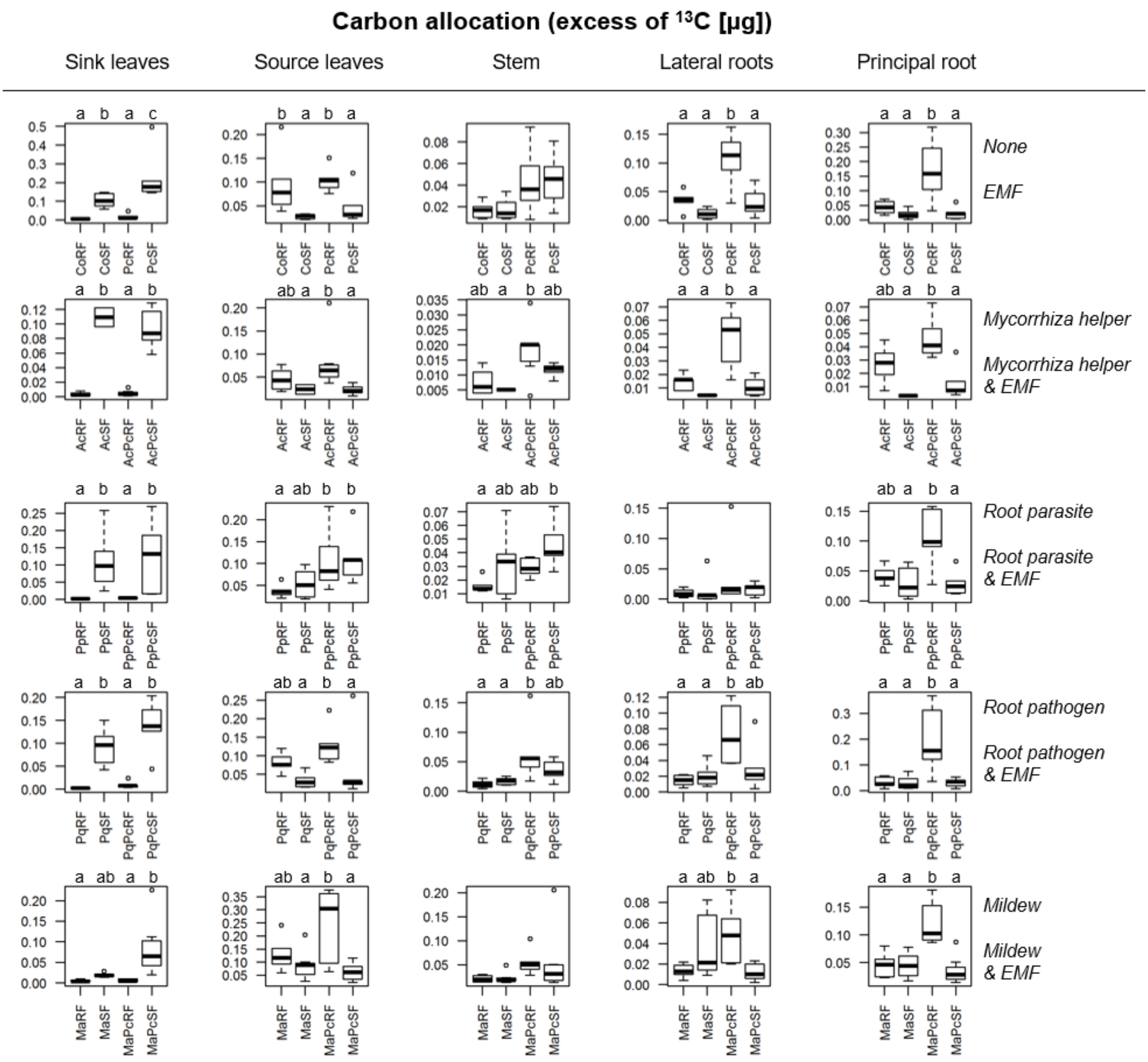
Carbon allocation (excess of 13C [µg])
Sink leaves
Source leaves
Stem
Lateral roots
Principal root
None
EMF
Mycorrhiza helper
Mycorrhiza helper & EMF
Root parasite
Root parasite & EMF
Root pathogen
Root pathogen & EMF
Mildew
Mildew & EMF

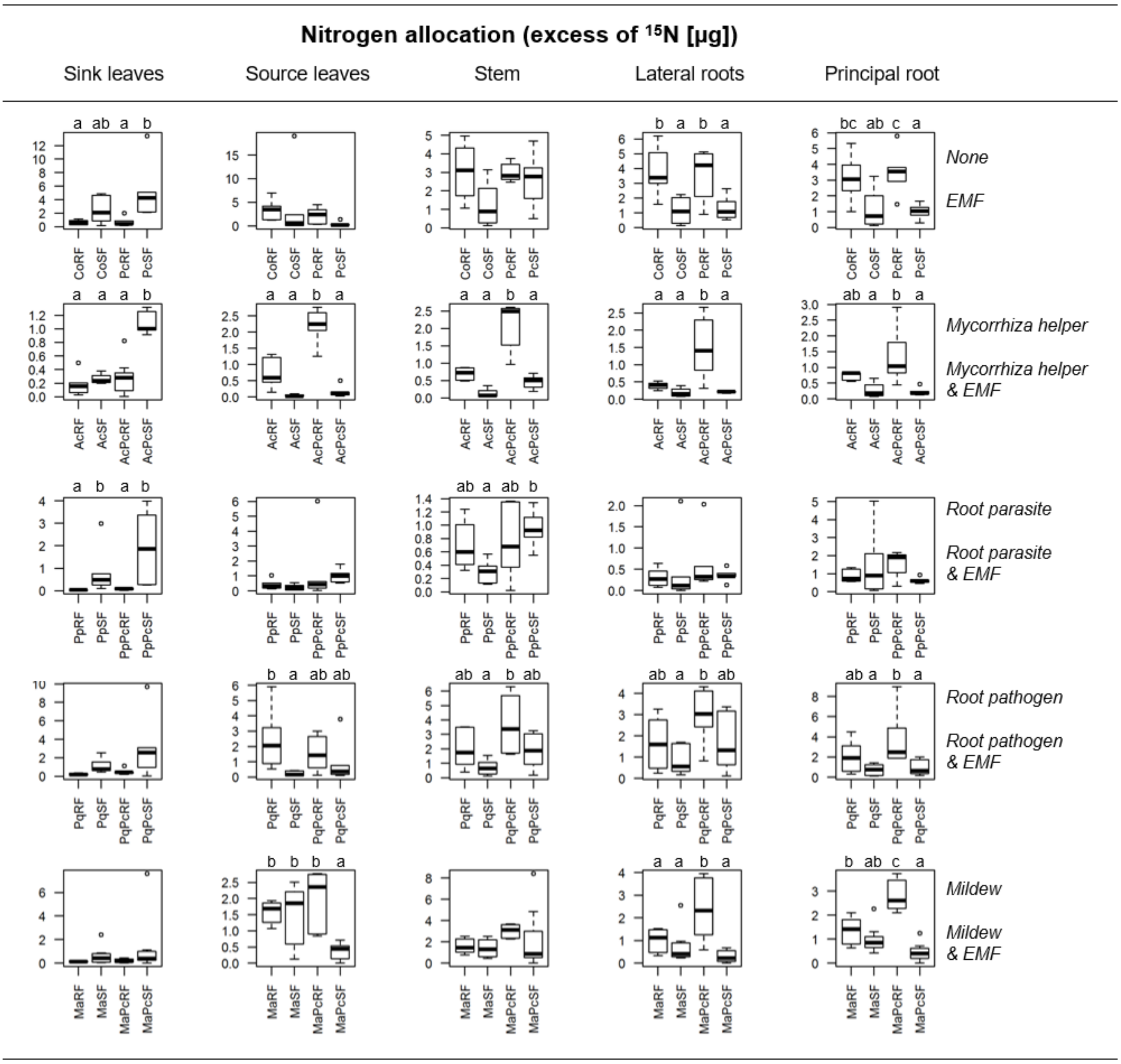

Nitrogen allocation (excess of ¹⁵N [µg])
Sink leaves
Source leaves
Stem
Lateral roots
Principal root
None
EMF
Mycorrhiza helper
Mycorrhiza helper & EMF
Root parasite
Root parasite & EMF
Root pathogen
Root pathogen & EMF
Mildew
Mildew & EMF
CoRF
CoSF
PcRF
PcSF
AcRF
AcSF
AcPcRF
AcPcSF
PpRF
PpSF
PpPcRF
PpPcSF
PqRF
PqSF
PqPcRF
PqPcSF
MaRF
MaSF
MaPcRF
MaPcSF

Supplementary Figure S4. Root-shoot ratios of dry weight, carbon and nitrogen allocation values in biotic interactions during root flush and shoot flush. Data and symbols as in Supplementary Figure S1. Different letters indicate difference according to linear model, ANOVA and Tukey test (P<0.05).

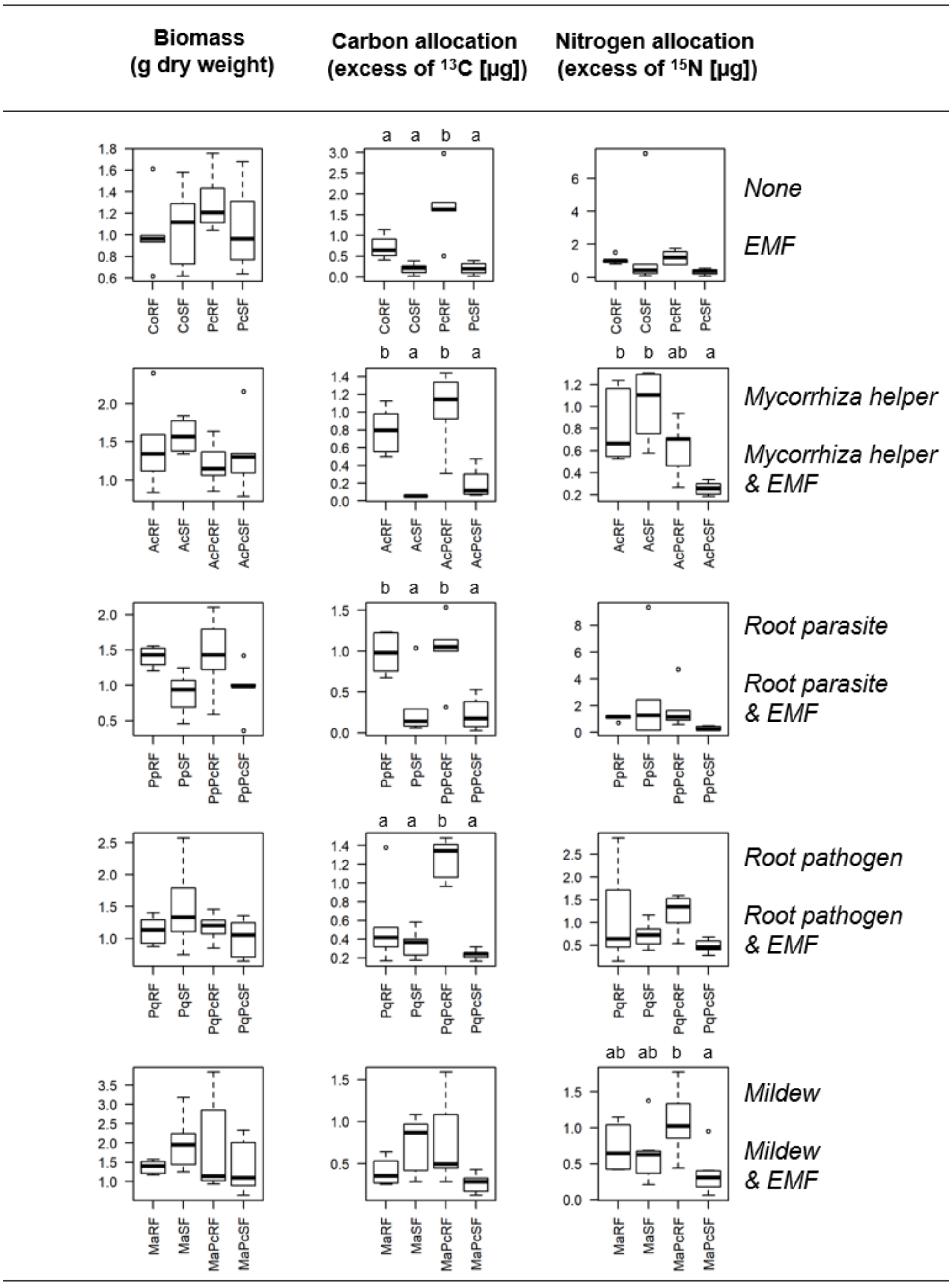

Supplement: Supplementary file 1 — Supplementary Information. [file 41598_2021_3132_MOESM1_ESM.pdf]
