# Supplementary material for: Ectomycorrhizal fungus supports endogenous rhythmic growth and corresponding resource allocation in oak during various below- and aboveground biotic interactions
Source: Sci Rep. 2021 Dec 8;11:23680. doi: 10.1038/s41598-021-03132-y (PMC8654951; doi:10.1038/s41598-021-03132-y)
Supplement: Supplementary file 3 — Supplementary Legends. [file 41598_2021_3132_MOESM3_ESM.docx]

**Supplementary Information**

**Supplementary Table S1.** Treatments of pedunculate oak DF159 (*Quercus robur* L.) microcuttings with five interacting organisms.

**Supplementary Table S2.** Numbers of samples obtained for the estimation of the distribution of biomass, carbon and nitrogen allocation during biotic interactions.

**Supplementary Table S3.** Effect of growth stage on the distribution of biomass, and carbon and nitrogen allocation in oak during biotic interactions, PERMANOVA analysis.

**Supplementary Table S4.** Effects of growth stage and additional treatment with mycorrhizal fungus on the distribution of plant biomass, carbon and nitrogen allocation at the level of plant organs.

**Supplementary Figure S1.** Schematic representation of the growth stages root flush and shoot flush in oak microcuttings.

**Supplementary Figure S2.** Principal components analysis of the distribution of biomass, recently fixed carbon and nitrogen in the sink and source leaves, stems, principal and lateral roots of oak microcuttings.

**Supplementary Figure S3.** Extent of biomass, recently fixed carbon and nitrogen in the individual organs of oak during root and shoot flush in oak microcuttings engaged in biotic interactions.

**Supplementary Figure S4.** Root-shoot ratios of dry weight, carbon and nitrogen allocation values in biotic interactions during root flush and shoot flush.
